# Variable improvement in whole grain consumption among youth by school lunch participation level in the United States: Findings from the 2007–2018 National Health and Nutrition Examination Survey

**DOI:** 10.1016/j.pmedr.2025.103017

**Published:** 2025-03-05

**Authors:** Mika Matsuzaki, Maria E. Acosta, Nancy Barba, Brisa N. Sánchez, Emma V. Sanchez-Vaznaugh

**Affiliations:** a Johns Hopkins Bloomberg School of Public Health. Department of International Health. Center for Human Nutrition. Baltimore, MD, United States; b San Francisco State University. Department of Public Health. San Francisco, CA, United States; c Drexel University Dornsife School of Public Health. Department of Epidemiology and Biostatistics. Philadelphia, PA, United States

**Keywords:** Whole grains, Adolescent health, Healthy, Hunger-free kids act 2010, Fiber, School nutrition

## Abstract

**Introduction:**

The Healthy, Hunger Free Kids Act (HHFKA) 2010 renewed food and nutrient requirements in school meals. Changes in whole grain consumption and fiber among youth may vary by school meal participation level.

**Methods:**

We analyzed cross-sectional data of five to seventeen year olds from the National Health and Nutrition Examination Survey across three policy periods (pre-policy (2007–2010), during the transition (2011–2014), and after HHFKA 2010 (2015–2018)). Weighted, multivariable regression models were fitted to estimate the association between policy periods and each of the dietary outcomes with interactions between policy periods and school meal participation levels, adjusting for sociodemographic characteristics.

**Results:**

Over 70 % of the children and adolescents participated in the school lunch program regularly in 2007–2018. Overall, there was an increasing trend in the whole grain consumption. At baseline, the group who did not participate in school lunch generally had a better nutritional profile including higher whole grain intake. However, the increase in whole grain intake after HHFKA 2010 among youth with regular participation, along with recent plateauing trends among infrequent participation groups, led to similar levels of whole grain intake across school meal participation groups. The amount of whole grain consumption among youth remained lower than the recommendation for all groups.

**Conclusion:**

After HHFKA 2010, the improvement in whole grain consumption among youth was variable by the levels of school meal participation. There is a need for additional measures of school-based interventions to promote whole grain and fiber consumption among youth.

## Highlights

• Prevention of nutrition-related chronic diseases is a public health priority.
• Whole grain intake may reduce risk of nutrition-related chronic diseases.
• The Healthy, Hunger-Free Kids Act 2010 required whole grain inclusion in school meals.
• Regular school lunch participants experienced improvement in whole grain intake after the policy.
• Whole grain intake continued to be suboptimal regardless of school meal participation levels.

## 1 Introduction

With the rising burden of nutrition-related chronic diseases, improvement in diet quality has become a public health priority globally. Cereal grains are one of the key sources of nutrients across food cultures (Ragaee et al., 2013). In comparison to refined grains, whole grains have more complex nutritional properties rich in vitamins, phytochemicals, and fibers (Okarter and Liu, 2010; Reynolds et al., 2020; Slavin, 2004). Previous studies have shown many beneficial effects of whole grain consumption (Jonnalagadda et al., 2011), including reduced risk of coronary heart diseases, cardiovascular disease, cancer, and mortality from all causes (Aune et al., 2016). Clinical trials have shown whole grains improve insulin sensitivities (Liese et al., 2003), lower total and LDL cholesterol levels (Hollænder et al., 2015), and lower inflammatory markers (Milesi et al., 2022; Kopf et al., 2018).

The United States Department of Agriculture (USDA) Dietary Guidelines for Americans recommends that at least half of the total grain intake to be whole grains (*U.S. Department of Agriculture and U.S. Department of Health and Human Services. Dietary Guidelines for Americans*, 2005). However, despite the national recommendation to increase whole grain consumption, research has shown that most children in the United States (U.S.) do not meet the recommended intake (Albertson et al., 2016; Tester, 2017; Fadeyev et al., 2021). A review of barriers to whole grain consumption among children and adolescents found poor availability of foods containing whole grains as one of the most commonly reported challenges (Meynier et al., 2020).

Over the past two decades, several efforts have attempted to increase the availability of whole grains and whole grain products, often as part of multi-component nutrition or health promotion programs. For example, since 2009, the Special Supplemental Nutrition Program for Women, Infants, and Children (WIC) revisions mandated that WIC-authorized stores must have whole grain bread and cereals available (Department of Agriculture, Food and Nutrition Service, 2007). Many of these programs to promote whole grain consumption found favorable changes in whole grain consumption among children and adolescents (Meynier et al., 2020; Oh et al., 2016; Oberle et al., 2020).

The Healthy, Hunger-Free Kids Act (HHFKA) 2010 made substantial changes to the nutrition standards for the National School Lunch and Breakfast Programs (NSLP/SBP), including requirements for providing 100 % whole grains and whole-grain food items and limiting sodium and saturated fat (United States Department of Agriculture, 2013). This policy is especially important given its reach to large populations and geographic scale. In 2021, 49.4 million students attended public Kindergarden-12 schools in the U.S, making schools an important venue for providing healthy meals for youth. Additionally, for youth from socially disadvantaged backgrounds, school lunch is an important source of nutrients. The Dietary Assessment Study analyzed menus and found improvement in the Healthy Eating Index 2010 score, which increased from 58 % to 82 % of the maximum scores after HHFKA 2010 (Gearan and Fox, 2020). Whole grain and refined grain component scores both improved by more than 20 percentage points in the same analysis. Recent reports also described increases in whole grain consumption among children and adolescents who participated in school lunch after HHFKA 2010 (Lin et al., 2023).

However, critical research gaps remain. Previous work focused on schoolchildren who participated in NSLP/SBP, but some children and adolescents do not participate in NSLP/SBP. In 2022, around 60 % of public school students received a free or reduced-price lunch through NSLP (Food Research and Action Center, 2024). Hence, we analyzed the National Health and Nutrition Examination Survey (NHANES) to examine the degree to which there may have been differences by the levels of school lunch participation in the trends of whole grain consumption among youth before and after HHFKA 2010 was implemented. Our analyses aimed to highlight both the impact of school nutrition policies and the potential limitations of the current policies in reaching all children and adolescents.

## 2 Methods

### 2.1 Study design and data collection

We used multiple waves of cross-sectional data from the National Health and Nutrition Examination Survey (NHANES) from 2007 to 08 to 2017–18. NHANES is a national survey that collects demographic, socioeconomic, dietary, and health-related data on adults and youth in a representative sample of the US population. The methods of data collection in NHANES have been published previously (National Center for Health Statistics, 2024). In the current analysis, the data files for each wave of data collection from 2007 to 2008 to 2017–2018 were appended. For children less than six years old, a knowledgeable adult responded to the survey questions; for ages six to eight, a proxy assisted the child in reporting intake information; for ages nine to eleven, the child responded with the assistance of an adult familiar with the child's intake; children aged twelve and older answered for themselves. Given that this analysis uses publicly available, secondary, de-identified data, this study does not meet the definition of human subject research and thus Institutional Review Board review was not needed.

### 2.2 Study sample

This analysis is restricted to children and adolescents of school age (five to seventeen years old), who reported whether they went to schools that serve lunch in cafeterias and had at least one day of dietary intake data collected during the weekdays. If children had two days of intake data on weekdays, we calculated the average of both days; otherwise, we used a single day. Individuals were excluded if their intake data were only available for weekends or missing. The total analytic sample size was 9421.

### 2.3 Exposures and outcomes

#### 2.3.1 Dietary intake

For dietary data, food and nutrient intake were determined using the United States Department of Agriculture Food and Nutrient Database for Dietary Studies (FNDDS), which contains the What We Eat in America food categories, food weights, ingredient lists, ingredient weights, and nutrient values (United States Department of Agriculture, 2022). Our models used whole grains (ounce equivalents), fiber intake(g), and total energy (kcal). Whole grain intake was determined from the Food Patterns Equivalents Database, which categorizes foods and beverages in the FNDDS into 37 Food Pattern components including Whole Grains, Refined Grains, and Total Grains (US Department of Agriculture, 2023). One ounce equivalent equals 16 g of whole grain ingredients.

#### 2.3.2 Participation in school lunch

School lunch participation was determined from the survey question “During the school year about how many times a week do you usually get a complete school lunch?” We classified children into three groups: zero days of school lunch participation, one to two days of participation, and three and more days of participation. We considered those participating in school lunch three days or more per week to be regular participants. All children and adolescents included in the analyses attended schools that served lunch (i.e., Yes to “Does your school serve school lunches?”).

#### 2.3.3 Time periods

The implementation of the HHFKA 2010 nutrition standards for school lunch began to take effect in the academic year 2012/13. Thus, we used 2007/08–2009/10 as the pre-HHFKA 2010, baseline period. We used 2011/12–2013/14 as the transition period as both of these two year cycles contain at least some of the transitional period after the beginning of the 2012/13 academic year (Jia et al., 2020) as the whole grain provision was incrementally introduced until the 2014/2015 academic year and by 2015, over 93 % of the schools reported to comply with the HHFKA 2010. We defined 2015/16–2017/18 as the post-policy implementation period.

#### 2.3.4 Additional variables

We included age, gender, race/ethnicity, ratio of family income to poverty threshold (United States Census Bureau, 2025), head of household education level and country of birth. Race/ethnic categories include Non-Hispanic White, Mexican American, Other Hispanic, Non-Hispanic Black and Other Race including multi-racial. Poverty income ratio was classified into four categories: 0–0.99, 1.00–1.99, 2.00–2.99, and ≥ 3.00 times the federal poverty level (FPL). We grouped the education levels of the head of household into three categories: college graduate or above, high school/General Education Development/Some college/associates degree and less than a high school education. Country of birth was used as a dichotomous variable indicating whether the child was born in the 50 U.S. states/Washington, District of Columbia or not.

### 2.4 Statistical analysis

The descriptive statistics were estimated for all variables for the overall sample and by the policy time periods: baseline (2007–2010), transition (2011–2014), and post HHFKA 2010 (2015–2018). Since we pooled the data across several two-year cycles, new weights were generated by dividing the sampling weights by the number of two year cycles included (National Center for Health Statistics, 2024). The mean weekday whole grain and fiber intakes were calculated for all the years. For whole grains and proportions of whole grains, the data were skewed, so medians and interquartile ranges were reported. We included the unweighted descriptive statistics in the appendix (Supplemental Material 1). For both weighted and unweighted descriptive statistics, we tested whether there were differences in characteristics across three policy periods by using either analyses of variance (continuous variables) or chi-squared tests (categorical variables).

Multivariable linear regression models were fitted to estimate the associations between school lunch participation, policy time periods, and each of the outcomes. Even though the distribution of whole grain intake was skewed, given the large sample size, non-transformed values were used to avoid potential biases in the model and prioritize interpretability of the results (Schmidt and Finan, 2018). The models were subsequently adjusted for age in years, gender, race/ethnicity, poverty to income ratio, education level of parents, country of birth, and total caloric intake. We tested for an interaction between school lunch participation and policy time periods to determine if the association between policy time period and dietary intake varied according to levels of participation in the school lunch. This allowed us to see the additional amounts of grains that are consumed after the policy among those who participated in the school lunch program regularly or infrequently compared to those who did not participate at all. We used additive interactions with continuous outcomes (VanderWeele and Knol, 2014). We also evaluated the interaction between policy periods and age groups (≤10, 11–14, and 15–17 years old) but found no clear evidence of interaction. For the regression models, we used sample weights for day 1 intake (Center for Diesease Control and Prevention, 2023). Analysis was conducted in October 2024 and January 2025 in R version 4.2.2.

## 3 Results

Over 70 % of the study population participated in the school lunch program three days or more in 2007–2018, although there was a slight decline in those who reported regular participation after the baseline period (Table 1). Survey-weighted mean caloric intake was about 1900 kcal (Standard Error (SE): 13.5). Overall, the estimated whole grain consumption was lower than the level recommended by the U.S. Dietary Guidelines for Americans (minimum 1.5 oz equivalents per day for 1000 kcal diet) (*U.S. Department of Agriculture and U.S. Department of Health and Human Services. Dietary Guidelines for Americans*, 2005; U.S. Department of Agriculture, 2010; U.S. Department of Agriculture, 2015; U.S. Department of Agriculture and U.S. Department of Health and Human Services, 2020), though the intake increased over time. About 4, 9, and 11 % of the total grain intake was estimated to be the median whole grain intake during the pre-policy, transitional, and post-policy periods respectively.

**Table 1:** Characteristics of the youth in the National Health and Nutrition Examination Survey in the United States (2007–2018). All numbers are percentages unless otherwise noted.

	HHFKA 2010 Policy Periods			Overall
	Pre-policy (2007–2010)	Transition (2011–2014)	Post-policy (2015–2018)	
Age (mean (SE) in years)	11.3 (0.1)	11.2 (0.1)	11.3 (0.1)	11.2 (0.1)

School lunch participation				
0 day/week	14.8	19.9	21.7	18.9
1 to 2 days/week	11	10.7	10.2	10.6
3 days or more/week	74.1	69.4	68.1	70.5

Gender				
Girls	51.7	50	49	50.2
Boys	48.3	50	51	49.8

Race/ethnicity				
Non-Hispanic White	58.2	53.3	50.6	53.9
Mexican American	13.8	15.6	15.7	15.1
Non-Hispanic Black	15.1	14.7	14.3	14.7
Other Hispanic	7.2	7.6	7.6	7.4
Other race	5.8	8.9	11.9	8.9

Poverty to income ratio				
0.00–0.99	24.1	25.9	22.1	24
1.00–1.99	23	24.9	25.5	24.5
2.00–2.99	14.5	15.2	15	14.9
≥3.00	38.4	34.1	37.4	36.5

Parental education				
Less than High School	21.1	19.8	16.6	19.1
High School/GED/Some college	52.7	52.8	54.6	53.4
College and above	26.2	27.4	28.8	27.5

Nativity				
Immigrant	6.1	5.5	4.2	5.3
U.S. Born	93.9	94.5	95.8	94.7

Food and nutrient intake (mean (SE) or median (IQR))				
Total calories (kcal) *	1918.6 (21.3)	1912.7(26.7)	1871.4 (20.9)	1900.7 (13.5)

Grains				
Total grain intake (ounce equivalents) **	6.73 (0.15)	6.98 (0.11)	6.98 (0.08)	6.9 (0.07)
Whole grain intake *** (ounce equivalents)	0.28 (0.89)	0.55 (1.27)	0.65 (1.48)	0.49 (1.23)
Percentage of whole grains per total grain intake ***	4.4 (14.5)	8.8 (19.8)	10.7 (24.3)	7.8 (19.5)
Refined grain intake (ounce equivalents)	6.12 (0.14)	6.10 (0.10)	5.97 (0.08)	6.06 (0.06)
Fiber (g) ***	13.5 (0.2)	14.7 (0.2)	14.6 (0.2)	14.3 (0.1)

HHFKA: Healthy, Hunger-Free Kids Act 2010; GED: General Educational Development; SE: Standard Errors; IQR: interquartile range. One ounce equivalent provides 16 g of whole grain ingredients. The numbers in Table 1 are weighted descriptive statistics. The unweighted descriptive statistics are included in the Supplemental Table 1.

FPL uses the definition set by the U.S. Census Bureau (United States Census Bureau, 2025).

* indicates *p* < 0.05; ** *p* < 0.01; *** *p* < 0.001 for tests for differences across time periods from the analyses of variance (continuous variables) or Chi squared tests (categorical variables).

The values for age, total grains, refined grains, dietary fiber, and calories are mean (standard deviations). Whole grains and proportions of whole grains per total grains are median (interquartile range).

**Fig. 1:**
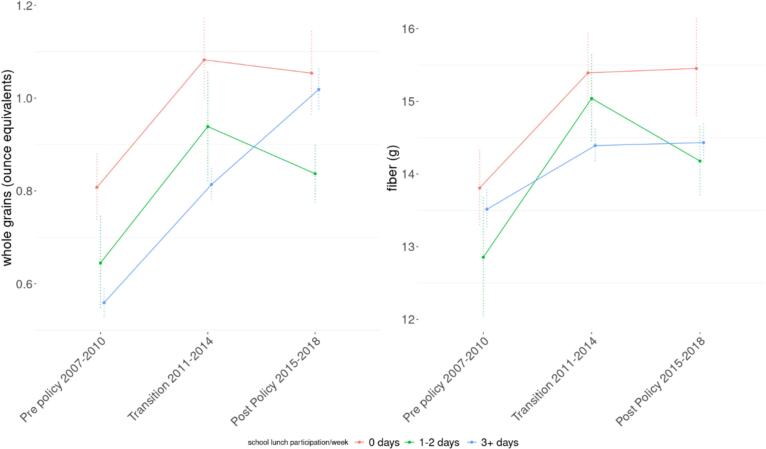
Changes in mean intake of whole grain and fiber in 2007–2018 by school lunch participation levels among children and adolescents before the Health, Hunger Free Kids Act (HHFKA) 2010, during the transitional period, and after HHFKA 2010. Weighted means and standard errors are shown.

We saw variable changes in whole grain consumption over time by the levels of school lunch program participation (Fig. 1). Before HHFKA 2010 was implemented, the group who reported not participating in school lunch had a better nutritional profile than more regular participants, with higher whole grain and fiber intake. All groups saw a rising trend in whole grain intake between the pre-policy and transitional period. However, this increase continued only among the youth who regularly participated in school lunch after HHFKA 2010. This led to the group's intake nearing the level of consumption among those who reported not participating in school lunch who had had a better nutritional profile at baseline. The groups who did not participate in school lunch regularly saw plateauing trends in whole grain consumption between the transitional period and after HHFKA 2010.

We tested the changes in whole grain and fiber intake by levels of school lunch participation in interaction models (Table 2). For whole grain intake, there was evidence of interactions by the levels of school lunch participation. Among the youth who regularly participated in school lunch, we saw clear evidence of an increase in whole grain intake in the period after the whole grain provision in the HHFKA 2010 was fully implemented. For fiber, we did not see clear evidence of interactions by school lunch participation level over time. Finally, despite the favorable changes in whole grain consumption among youth, the amounts of whole grains consumed was only one ounce equivalent in the most recent study period.

**Table 2:** Multivariable linear regression models examining associations between policy periods and whole grain or fiber intake among youth in the United States (2007–2018).

	Whole grains (ounce equivalents^1^)		Fiber (grams)	
	**Coefficients (95 % CI)**	***p*-value**	**Coefficients (95 % CI)**	**p-value**
**Policy time period**^**2**^				
Transition period (2011–2014)	0.26 (0.05 to 0.47)	0.02	1.46 (0.43 to 2.48)	<0.01
Post policy (2015–2018)	0.21 (−0.01 to 0.43)	0.06	1.37 (0.03 to 2.71)	0.04

**Weekly school lunch participation**^**3**^				
1–2 days	−0.19 (−0.40 to 0.02)	0.07	−0.81 (−2.35 to 0.72)	0.29
3 days or more	0.24 (−0.40 to −0.07)	<0.01	−1.08 (−1.83 to −0.33)	<0.01

**Interaction between time period and school lunch participation**^**4**^				
Transition:1–2 days	0.00 (−0.36 to 0.35)	1.00	−0.62 (−2.56 to 1.32)	0.53
Post policy:1–2 days	−0.03 (−0.35 to 0.29)	0.85	−0.61 (−2.64 to 1.42)	0.55
Transition:3+ days	0.00 (−0.23 to 0.23)	0.99	−0.40 (−1.47 to 0.67)	0.46
Post policy:3+ days	0.27 (0.00 to 0.53)	0.05	0.02 (−1.42 to 1.46)	0.98

The model was adjusted for age in years, gender, race/ethnicity, poverty to income ratio as defined by the U.S. Census Bureau (United States Census Bureau, 2025), education level of parents, country of birth (U.S. or other), and total caloric intake.

1. One ounce equivalent provides 16 g of whole grain ingredients.

2. Reference category was the period before HHFKA 2010.

3. Reference category was no participation.

4. Reference value was the period before HHFKA 2010 and no school lunch participation.

## 4 Discussion

After HHFKA 2010 went into effect, we found evidence of increases in whole grain consumption among the youth who regularly participated in the National School Lunch Program. However, among the youth with infrequent or no school lunch participation, despite their whole grain intake being highest before the policy went into effect, there was a plateauing trend in whole grain consumption in recent years. Regardless of the school lunch participation levels, overall, the youth on average had low daily intake of whole grains, at or less than one ounce equivalent, in comparison to six ounce equivalents in refined grains. To our knowledge, this is the first study to examine the potential population-level impact of the national school nutrition policy on whole grain intake with consideration to the varying levels of school lunch participation. The findings highlight the importance of whole grain inclusion in the nutrition policies for school meals as well as the need for additional systemic interventions beyond school meals to improve whole grain consumption among all children and adolescents.

Grains constitute a key component of meals across food cultures. Whole grains contain all parts of the grain structure including germ, bran, and endosperm, offering more fiber, vitamins, minerals, and phytochemicals than refined grains (Okarter and Liu, 2010; Reynolds et al., 2020; Jonnalagadda et al., 2011). Refinement of grains leads to loss of one or more of these structural parts, resulting in lower nutrient availability. Studies have repeatedly shown beneficial effects of whole grain intake in association with nutrition-related chronic diseases like type 2 diabetes and cardiovascular diseases (Aune et al., 2016; Aune et al., 2013; Marshall et al., 2020). Given clear evidence of the beneficial impact of whole grains over refined grains, since 2005, the Dietary Guidelines for Americans has included a recommendation that more than half of the grain intake should be from whole grains (*U.S. Department of Agriculture and U.S. Department of Health and Human Services. Dietary Guidelines for Americans*, 2005). In 2010, HHFKA 2010 was passed and mandated inclusion of whole grains in school lunch. Previous studies have suggested increases in whole grain consumption after HHFKA 2010 among youth and this increase was largely attributed to intake in school lunch.

Our results on the overall increase in whole grain consumption after HHFKA 2010 are in line with previous studies on whole grain inclusion from menu analyses or NSLP participants' consumption of whole grains (Gearan and Fox, 2020; Lin et al., 2023; Lin et al., 2019). However, given that only 60 % of children have participated in NSLP in recent years (Food Research and Action Center, 2024), this study contributes to the literature by evaluating potential differences in levels of improvement in whole grain intake by school lunch participation. This study's findings suggest that intake of whole grains increased after HHFKA 2010 was introduced, but only among children who regularly participate in the school lunch program. One potential explanation for this observation is that children who participate more regularly may have more limited exposure to whole grains outside of schools and the increase in whole grain availability in schools may have contributed to the clear increase in whole grain consumption. The group of students who reported not participating in NSLP saw an upward trend in whole grain consumption in the transitional period but this trend plateaued in more recent years. These students may have other opportunities to consume whole grains, such as at home. However, our results suggest that these other sources were not enough to reach the recommended levels of daily intake and those students did not experience additional exposure from the improved availability of whole grains at school following HHFKA 2010's implementation. There is a need for future studies to identify other ways to use schools as a venue to improve whole grain consumption among all youth, ideally with a lasting impact beyond secondary school years.

### 4.1 Implications for future policies and research

The present study provides evidence for the favorable changes in whole grain consumption following HHFKA 2010, especially among regular participants of the NSLP. Our findings also highlight the need for additional interventions to reach all children and adolescents because whole grain consumption among youth continues to be well below the recommended levels. Our study results also imply that, if the increase in whole grain consumption seen among students with regular participation in school lunch is dependent on the school lunch programs, there would be a substantial decline in whole grain consumption after the compulsory education ends, potentially reversing the favorable changes the students experienced in school. Stronger nutrition education and supportive food environments in general are needed to develop lifelong healthy eating habits within and beyond school settings. Schools may also want to involve families, communities, and stakeholders in school food systems in improving availability of and access to whole grains. The sustainability of healthy dietary behaviors and access to and availability of high quality foods needs to be prioritized in future school nutrition policies.

Globally, national school lunch programs influence diet quality of a large number of children and adolescents as many countries have mandatory education. There are currently few countries that include a specific provision on whole grains in school lunch. Our findings suggest that there may be a potential beneficial impact of whole grain introduction in school lunch on whole grain intake among youth, especially for those who regularly participate in school lunch programs. In countries without a specific provision on whole grains in school lunch, our findings provide evidence for policy-makers to consider potential introduction of similar policies for school meals in the future.

### 4.2 Strengths and Limitations

The current study is the first to provide evidence on the potential impact of HHFKA 2010 on whole grain consumption by the levels of school participation. Unlike aggregated analyses, this approach allowed us to compare children who were more likely to be impacted by HHFKA 2010 vs. those who may have been less affected. We were limited in our ability to directly measure consumption of whole grains in schools and other settings as the consumption is aggregated in the NHANES dataset. While the trend analyses over three policy periods support the favorable policy impact, we cannot eliminate the possibility of other factors contributing to the variable changes in whole grain consumption in the same time frame. HHFKA 2010 also affected the School Breakfast Program, which often includes whole grain-based items, but in this study, we could not examine whether whole grain consumption differed by the levels of participation of the School Breakfast Program.

## 5 Conclusion

This study found promising evidence of the effectiveness of school nutrition policies to improve whole grain consumption among youth who participated regularly in NSLP. However, we also found low levels of whole grain intake among youth overall, as well as a concerning trend of the lack of improvement among youth who do not participate in school lunch regularly. Additional measures are needed to improve whole grain consumption among youth.

## CRediT authorship contribution statement

**Mika Matsuzaki:** Writing – review & editing, Writing – original draft, Visualization, Formal analysis, Conceptualization. **Maria E. Acosta:** Writing – review & editing, Validation, Formal analysis, Conceptualization. **Nancy Barba:** Writing – review & editing, Validation, Formal analysis, Conceptualization. **Brisa N. Sánchez:** Writing – review & editing, Supervision, Methodology, Funding acquisition. **Emma V. Sanchez-Vaznaugh:** Writing – review & editing, Supervision, Methodology, Funding acquisition, Conceptualization.

## Funding

Mika Matsuzaki was supported by the National Heart, Lung, and Blood Institute (K01HL165465). Brisa N. Sánchez and Emma V. Sanchez-Vaznaugh were supported by the National Insisute of Minority Health and Health Disparities (R01MD017687) and Eunice Kennedy Shriver National Institute of Child Health and Human Development (R01HD111169).

## Declaration of competing interest

The authors declare that they have no known competing financial interests or personal relationships that could have appeared to influence the work reported in this paper.

## Data Availability

NHANES is a publicly available dataset.

## Appendix A Supplementary data

Supplementary material Table 1 Characteristics of the youth (5-17 years old) in the United States by the Healthy, Hunger-Free Kids Act 2010 policy time period in the National Health and Nutrition Examination Survey (2007-2018). Image 1

## References

[bb0005] Albertson A.M., Reicks M., Joshi N., Gugger C.K. (2016). Whole grain consumption trends and associations with body weight measures in the United States: results from the cross sectional National Health and nutrition examination survey 2001–2012. Nutr. J..

[bb0010] Aune D., Norat T., Romundstad P., Vatten L.J. (2013). Whole grain and refined grain consumption and the risk of type 2 diabetes: a systematic review and dose-response meta-analysis of cohort studies. Eur. J. Epidemiol..

[bb0015] Aune D., Keum N., Giovannucci E., Fadnes L.T., Boffetta P., Greenwood D.C. (2016). Whole grain consumption and risk of cardiovascular disease, cancer, and all cause and cause specific mortality: systematic review and dose-response meta-analysis of prospective studies. BMJ.

[bb0020] Center for Diesease Control and Prevention (2023). NHANES Tutorials - Weighting Module. In: National Center for Health Statistics [Internet]. https://wwwn.cdc.gov/nchs/nhanes/tutorials/weighting.aspx.

[bb0025] Department of Agriculture, Food and Nutrition Service (2007). https://www.federalregister.gov/documents/2024/04/18/2024-07437/special-supplemental-nutrition-program-for-women-infants-and-children-wic-revisions-in-the-wic-food.

[bb0030] Fadeyev K., Nagao-Sato S., Reicks M. (2021). Nutrient and food group intakes among U.S. children (2-5 years) differ by family income to poverty ratio, NHANES 2011-2018. Int. J. Environ. Res. Public Health.

[bb0035] Food Research and Action Center (2024). National School Lunch Program. In: Food Research & Action Center. https://frac.org/programs/national-school-lunch-program.

[bb0040] Gearan E.C., Fox M.K. (2020). Updated nutrition standards have significantly improved the nutritional quality of school lunches and breakfasts. J. Acad. Nutr. Diet..

[bb0045] Hollænder P.L., Ross A.B., Kristensen M. (2015). Whole-grain and blood lipid changes in apparently healthy adults: a systematic review and meta-analysis of randomized controlled studies123. Am. J. Clin. Nutr..

[bb0050] Jia J., Moore L.L., Cabral H., Hanchate A., LaRochelle M.R. (2020). Changes to dietary and health outcomes following implementation of the 2012 updated US Department of Agriculture school nutrition standards: analysis using National Health and nutrition examination survey, 2005-2016. Public Health Nutr..

[bb0055] Jonnalagadda S.S., Harnack L., Hai Liu R., McKeown N., Seal C., Liu S. (2011). Putting the whole grain puzzle together: health benefits associated with whole grains—summary of American Society for Nutrition 2010 satellite Symposium1–3. J. Nutr..

[bb0060] Kopf J.C., Suhr M.J., Clarke J., Eyun S., Riethoven J.-J.M., Ramer-Tait A.E. (2018).

[bb0065] Liese A.D., Roach A.K., Sparks K.C., Marquart L., D’Agostino R.B., Mayer-Davis E.J. (2003). Whole-grain intake and insulin sensitivity: the insulin resistance atherosclerosis Study2. Am. J. Clin. Nutr..

[bb0070] Lin B.-H., Guthrie J.F., Smith T.A. (2019). Dietary guidance and new school meal standards: Schoolchildren’s whole grain consumption over 1994–2014. Am. J. Prev. Med..

[bb0075] Lin B.-H., Smith T., Guthrie J. (2023). Trends in U.S. Whole-Grain Intakes 1994–2018: The Roles of Age, Food Source, and School Food.

[bb0080] Marshall S., Petocz P., Duve E., Abbott K., Cassettari T., Blumfield M. (2020). The effect of replacing refined grains with whole grains on cardiovascular risk factors: a systematic review and Meta-analysis of randomized controlled trials with GRADE clinical recommendation. J. Acad. Nutr. Diet..

[bb0085] Meynier A., Chanson-Rollé A., Riou E. (2020). Main factors influencing whole grain consumption in children and adults—a narrative review. Nutrients.

[bb0090] Milesi G., Rangan A., Grafenauer S. (2022). Whole grain consumption and inflammatory markers: a systematic literature review of randomized control trials. Nutrients.

[bb0095] National Center for Health Statistics (2024). NHANES Survey Methods and Analytic Guidelines. In: Centers for Disease Control and Prevention [Internet]. https://wwwn.cdc.gov/nchs/nhanes/analyticguidelines.aspx.

[bb0100] Oberle M.M., Freese R., Shults J., Stallings V.A., Virudachalam S. (2020). Impact of the 2009 WIC food package changes on maternal dietary quality. J. Hunger Environ. Nutr..

[bb0105] Oh M., Jensen H.H., Rahkovsky I. (2016). Did revisions to the WIC program affect household expenditures on whole grains?. Appl. Econ. Perspect. Policy.

[bb0110] Okarter N., Liu R.H. (2010). Health benefits of whole grain phytochemicals. Crit. Rev. Food Sci. Nutr..

[bb0115] Ragaee S., Gamel T., Seethraman K., Abdel-Aal E.-S.M. (2013). Handbook of Plant Food Phytochemicals.

[bb0120] Reynolds A.N., Akerman A.P., Mann J. (2020). Dietary fibre and whole grains in diabetes management: systematic review and meta-analyses. PLoS Med..

[bb0125] Schmidt A.F., Finan C. (2018). Linear regression and the normality assumption. J. Clin. Epidemiol..

[bb0130] Slavin J. (2004). Whole grains and human health. Nutr. Res. Rev..

[bb0135] Tester J.M. (2017). Recent uptrend in whole-grain intake is absent for low-income adolescents, National Health and nutrition examination survey, 2005–2012. Prev. Chronic Dis..

[bb0140] U.S. Department of Agriculture (2010).

[bb0145] U.S. Department of Agriculture (2015).

[bb0150] U.S. Department of Agriculture and U.S. Department of Health and Human Services (2020 Dec). http://DietaryGuidelines.gov.

[bb0155] (2005). U.S. Department of Agriculture and U.S. Department of Health and Human Services. Dietary Guidelines for Americans.

[bb0160] United States Census Bureau (2025). How the Census Bureau Measures Poverty. https://www.census.gov/topics/income-poverty/poverty/guidance/poverty-measures.html.

[bb0165] United States Department of Agriculture (2013). National School Lunch Program and school breakfast program: nutrition standards for all foods sold in school as required by the healthy, hunger-free kids act of 2010. Fed. Regist..

[bb0170] United States Department of Agriculture (2022). Food and Nutrient Database for Dietary Studies. 21 Oct. https://www.ars.usda.gov/northeast-area/beltsville-md-bhnrc/beltsville-human-nutrition-research-center/food-surveys-research-group/docs/fndds/.

[bb0175] US Department of Agriculture. FPED methodology : USDA ARS. In: Food Surveys Research Group: Beltsville, MD [Internet]. 19 Jul 2023 [cited 18 Sep 2023]. Available: https://www.ars.usda.gov/northeast-area/beltsville-md-bhnrc/beltsville-human-nutrition-research-center/food-surveys-research-group/docs/fped-methodology/.

[bb0180] VanderWeele T.J., Knol M.J. (2014).

