# Supplementary material for: Variable improvement in whole grain consumption among youth by school lunch participation level in the United States: Findings from the 2007–2018 National Health and Nutrition Examination Survey
Source: Prev Med Rep. 2025 Mar 5;53:103017. doi: 10.1016/j.pmedr.2025.103017 (PMC11957667; doi:10.1016/j.pmedr.2025.103017)
Supplement: Supplementary file 1 — Supplementary material Table 1 Characteristics of the youth (5-17 years old) in the United States by the Healthy, Hunger-Free Kids Act 2010 policy time period in the National Health and Nutrition Examination Survey (2007-2018). [file mmc1.docx]

**Supplemental Material 1. Characteristics of the youth (5-17 years old) in the United States by the Healthy, Hunger-Free Kids Act 2010 policy time period in the National Health and Nutrition Examination Survey (2007-2018). All values are crude (unweighted) percentages unless otherwise noted.**

|  | **Policy Periods** | | | **Overall** |
| --- | --- | --- | --- | --- |
|  | **Pre policy**  **(2007-10)** | **Transitional (2011-14)** | **Post policy**  **(2015-18)** |  |
| **Age (mean (SD) in years) **** | 10.9 (3.6) | 10.8 (3.6) | 11.1 (3.6) | 10.9 (3.6) |
| **School lunch participation ***** |  |  |  |  |
| 0 day/week | 12.2 | 16.7 | 15.9 | 14.9 |
| 1 to 2 days/week | 7.7 | 9.1 | 8.6 | 8.5 |
| 3 days or more/week | 80.1 | 74.2 | 75.5 | 76.6 |
| **Gender** |  |  |  |  |
| Girls | 49.3 | 49.3 | 50.2 | 49.6 |
| Boys | 50.7 | 50.7 | 49.8 | 50.4 |
| **Race/ethnicity ***** |  |  |  |  |
| Non-Hispanic White | 31.1 | 24.9 | 29.3 | 28.3 |
| Mexican American | 26.4 | 20.8 | 19.8 | 22.4 |
| Non-Hispanic Black | 24.7 | 28.6 | 24.1 | 25.9 |
| Other Hispanic | 12.3 | 10.3 | 9.7 | 10.8 |
| Other race/ethnicity | 5.5 | 15.4 | 17.0 | 12.5 |
| **Poverty to income ratio ***** |  |  |  |  |
| 0.00-0.99 | 32.5 | 35.1 | 27.7 | 32.0 |
| 1.00-1.99 | 28.3 | 27.7 | 31.1 | 28.9 |
| 2.00-2.99 | 13.8 | 11.8 | 15.6 | 13.6 |
| ≥3.00 | 25.5 | 25.4 | 25.6 | 25.5 |
| **Parental education ***** |  |  |  |  |
| Less than High School | 30.3 | 25.2 | 20.8 | 25.6 |
| High School/GED/Some college | 52.1 | 52.1 | 57.1 | 53.6 |
| College and above | 17.6 | 22.7 | 22.0 | 20.8 |
| **Nativity *** |  |  |  |  |
| Immigrant | 7.4 | 8 | 6.3 | 7.3 |
| U.S. Born | 92.6 | 92 | 93.7 | 92.7 |
| **Food group and nutrients (mean (SD) and median (IQR))** | | | | |
| Calories (kcal) * | 1885.2 (716.7) | 1886.8 (717.0) | 1845.8 (741.4) | 1873.95 (724.43) |
| **Grains** |  |  |  |  |
| Total Grains (ounce equivalents) *** | 6.57 (3.41) | 6.94 (3.54) | 6.87 (3.57) | 6.79 (3.51) |
| Whole Grains (ounce equivalents) *** | 0.26 (0.83) | 0.52 (1.27) | 0.61 (1.43) | 0.45 (1.19) |
| Percentage of whole grains per total grain intake *** | 4.4 (13.8) | 8.4 (19.6) | 10.0 (24.1) | 7.3 (18.7) |
| Refined Grains (ounce equivalents) | 5.99 (3.29) | 6.09 (3.42) | 5.88 (3.49) | 6.00 (3.40) |
| Dietary Fiber (g) *** | 13.5 (6.8) | 14.6 (7.3) | 14.4 (7.4) | 14.2 (7.2) |

SD: standard deviations; U.S.: United States of America; GED: General Educational Development; IQR: interquartile range

Poverty is the federal poverty level set by the U.S. Census Bureau.

All values are unweighted. The total analytic sample size was 9,421. For pre-policy, transitional period, and post-policy, 3,221, 3,377, and 2,823 participants were included.

* indicates p<0.05; ** p<0.01; *** p<0.001 for differences between pre-policy and transition or post-policy periods from analysis of variance (continuous variables) or Chi squared tests (categorical variables).

The values for age, total grains, refined grains, dietary fiber, and calories are mean (standard deviations). Whole grains and proportions of whole grains per total grains are median (interquartile range).
